# Pituitary macroadenoma apoplexy as a rare complication of Bruton tyrosine kinase inhibitor in chronic lymphoid leukaemia

**DOI:** 10.1186/s41016-023-00345-0

**Published:** 2023-10-24

**Authors:** Aysha Gomaa, Robert Skelly

**Affiliations:** 1 East Sussex Healthcare NHS Trust, Eastbourne, UK; 2 East Suffolk and North Essex NHS Foundation Trust, Colcchester, UK

**Keywords:** Pituitary apoplexy, Pituitary macroadenoma, Chronic lymphocytic leukaemia, Chemotherapy

## Abstract

**Background:**

Pituitary apoplexy is a neurosurgical emergency and is a known yet rare complication of pituitary macroadenoma. Patients typically present with visual field defects, headache and altered sensorium. There are multiple risk factors for this complication and a thorough drug history is essential to exclude iatrogenic causes of disease. We present an extremely rare case of newly diagnosed pituitary insufficiency unveiled by ibrutinib therapy (a Bruton tyrosine kinase inhibitor). Furthermore, after initial withdrawal of ibrutinib because of the erroneous diagnosis of Syndrome of Inappropriate Antidiuretic Hormone Secretion (SIADH), its re-administration led to the development of classical pituitary apoplexy 4 months after treatment was restarted.

**Case presentation:**

A male patient in his 60s with a background of chronic lymphocytic leukaemia (CLL) on ibrutinib and venetoclax presents with acute confusion and deranged electrolytes. He is found to be hyponatraemic and is diagnosed with Syndrome of Inappropriate Antidiuretic Hormone Secretion (SIADH) and treated with fluid restriction. He represents again 3 weeks later with hyponatraemia and further investigations reveal pituitary insufficiency and macroadenoma. He was restarted on ibrutinib and venetoclax at the time of discharge.

Four months later, he presents with sudden retro-orbital headache associated with vomiting. Clinical findings include cranial nerve III, IV and XI palsy. Humphrey’s visual field examination revealed a left visual field index (VFI) of only 1% while the right was 64% with temporal hemianopia. Both pupils were mid-dilated and poorly reactive to light. MRI pituitary with contrast showed features of pituitary apoplexy and optic nerve compression. He was urgently referred to the neurosurgical team and underwent an emergency trans-sphenoidal hypophysectomy with circumferential excision of the macroadenoma. Post-operative recovery was uneventful with marked improvement in vision bilaterally.

The patient was restarted on ibrutinib and venetoclax 2 weeks post-operatively. Approximately 1 year post-treatment, he remains in radiological, clinical and biochemical remission from CLL and all medications have been withdrawn.

**Conclusions:**

This is a unique and rare case of pituitary macroadenoma apoplexy following the commencement of ibrutinib for CLL. Central nervous system haemorrhage is a rare side effect of ibrutinib due to its platelet dysfunction effects. A thorough assessment is required to assess the risks and benefits of using ibrutinib in patients with pituitary macroadenoma to avoid serious complications.

## Background

Pituitary apoplexy is a neurosurgical emergency. It is a known yet rare complication of pituitary macroadenoma [1]. Patients typically present with visual field defects, headache and altered sensorium [1]. There are multiple risk factors for this complication and a thorough drug history is essential to exclude iatrogenic causes of disease [1]. We present an extremely rare case of newly diagnosed pituitary insufficiency unveiled by ibrutinib therapy (a Bruton tyrosine kinase inhibitor). Furthermore, after the initial withdrawal of treatment of ibrutinib because of the erroneous diagnosis of Syndrome of Inappropriate Antidiuretic Hormone Secretion (SIADH), its re-administration led to the development of classical pituitary apoplexy 4 months after treatment was restarted. There is only one similar reported case in the United States and it is unclear if the patient was restarted on ibrutinib.

## Case presentation

**Fig. 1:**
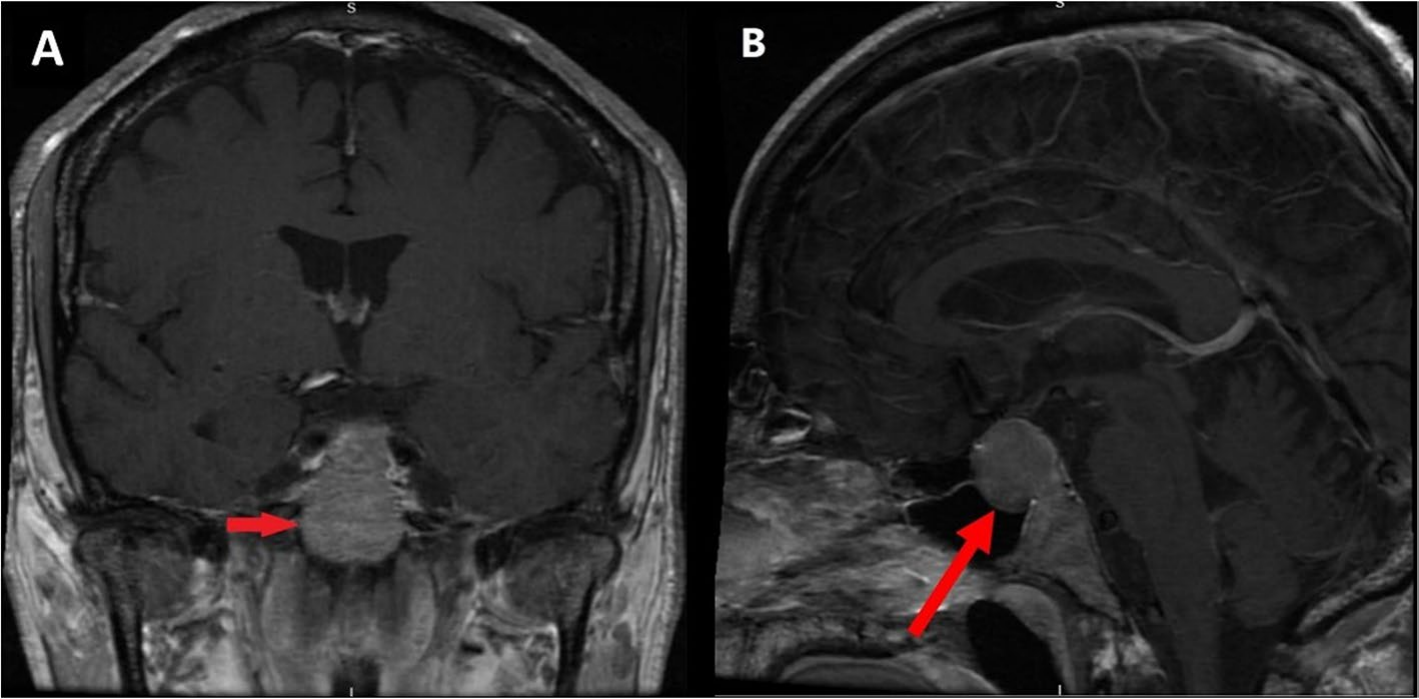
Initial MRI pituitary with contrast. **A** T1 coronal MRI showing pituitary macroadenoma (2 × 1.9 × 1.5 cm). **B** T1 sagittal MRI showing pituitary macroadenoma

A male patient in his 60s with progressive chronic lymphocytic leukaemia (CLL) despite standard therapy. A combination of ibrutinib and venetoclax have been used in patients with refractory CLL [2] as in this case. His past medical history included trisomy 12 and primary hypothyroidism. A month later he presented to the Emergency Department (ED) with confusion and hyponatraemia. Paired serum and urine sodium and osmolality showed a serum sodium 120 mmol/L, urine sodium 77 mmol/L, serum osmolality 265 mOsm/kg and urine osmolality 792 mOsm/kg. He was diagnosed with Syndrome of Inappropriate Antidiuretic Hormone Secretion (SIADH) and both ibrutinib and venetoclax were stopped during the admission. Hyponatraemia resolved following fluid restriction and both chemotherapy agents were restarted on discharge. Three weeks later, he presented with symptoms of nausea, diarrhoea and hypotension. Bloods again demonstrated hyponatraemia with sodium of 122 mmol/L. A full pituitary profile revealed pituitary insufficiency. Serum 09:00 cortisol was 15 nmol/L with adrenocorticotropic hormone (ACTH) of 11 ng/L. Other blood results included: testosterone 0.42 nmol/L, luteinising hormone (LH) 13 IU/L, follicular stimulating hormone (FSH) 1.7 IU/L, thyroid stimulating hormone (TSH) 0.12 mU/L, thyroxine 12 pmol/L and serum insulin-like growth factor 1 (IGF-1) was normal. Treatment with hydrocortisone was commenced. MRI pituitary revealed a pituitary macroadenoma as shown in Fig. 1. He was discharged feeling “like a new man” on hydrocortisone 15/5/5 mg daily. The haematology team were anxious to stop his treatment and ibrutinib was commenced at a lower dose.

**Fig. 2:**
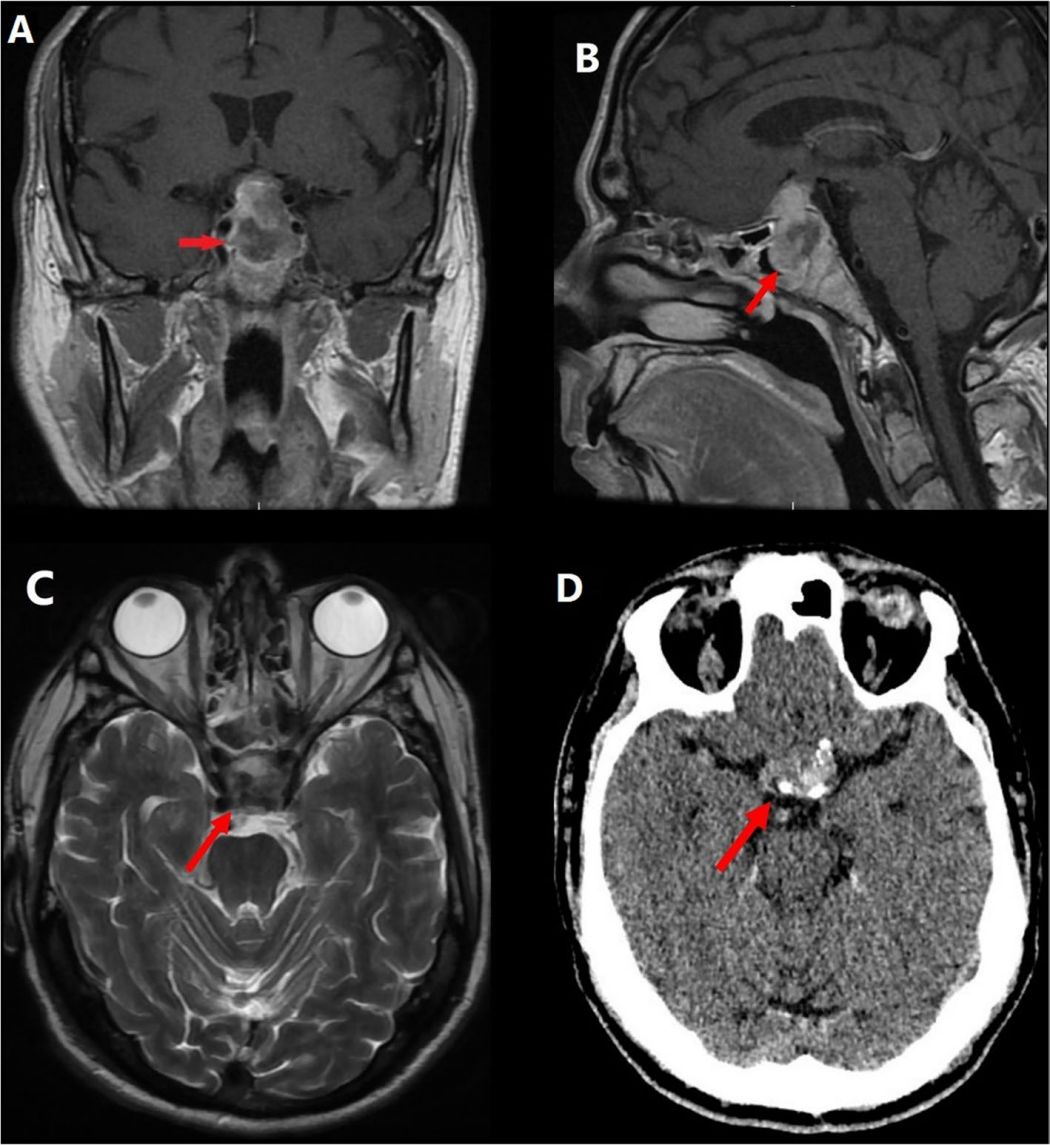
Subsequent imaging 3 months later demonstrating pituitary apoplexy. **A** T1 coronal MRI showing pituitary macroadenoma (2.6 × 2 × 2.3 cm) with evidence of apoplexy and stretching and compression of the optic chiasm. **B** T1 sagittal MRI with contrast. **C** T2 axial MRI without contrast. **D** non-contrast CT head

**Fig. 3:**
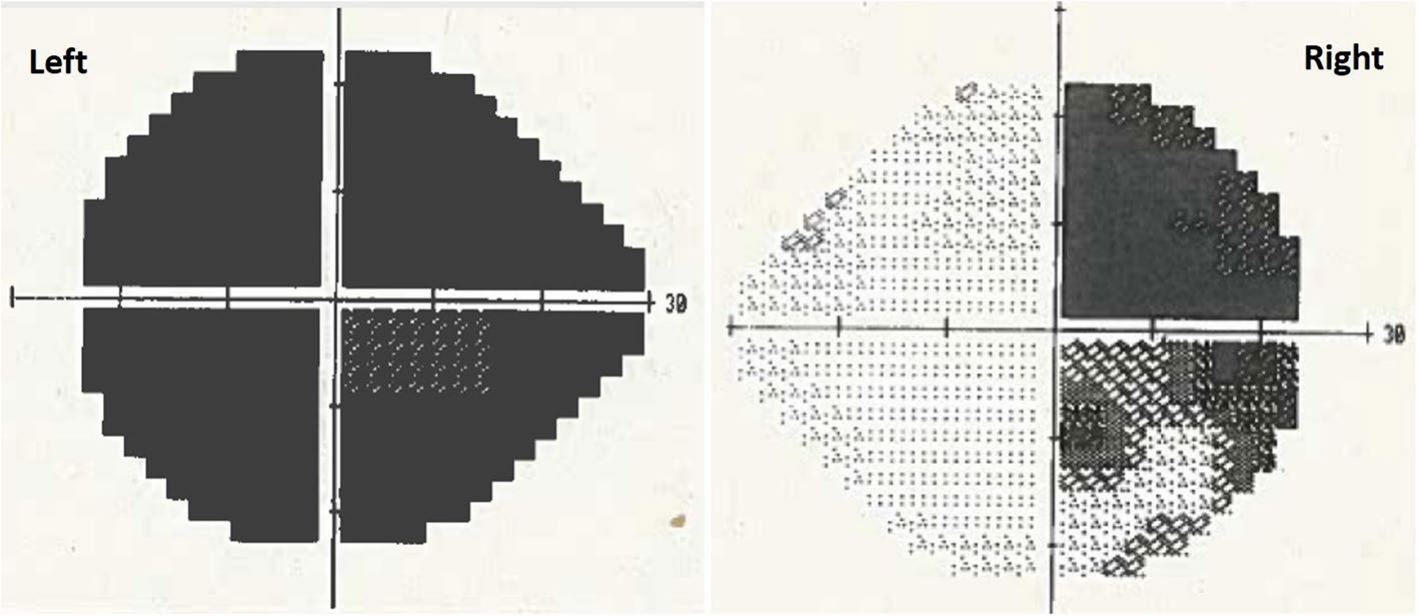
Humphrey’s visual field test results. Left: Visual field index (VFI) of the left eye is 1% with features of complete visual loss suggestive of optic nerve compression. Right: VFI of the right eye is 64% with features of temporal hemianopia

Four months later, the patient presented with a 3-day history of sudden retro-orbital headache associated with vomiting, lethargy and anorexia. Examination findings included left cranial nerve III, IV and XI palsy, left-sided ptosis with blurred vision and diplopia. Left eye examination demonstrated blurred vision and diplopia. Both pupils were mid-dilated and poorly reactive to light; this was more marked on the left. Visual field test by confrontation revealed right temporal hemianopia and near-complete vision loss in the left eye. An urgent CT head followed by an MRI pituitary revealed a 30% increase in the size of the tumour as well as features suggestive of pituitary apoplexy as shown in Fig. 2. The tumour was invading the left cavernous sinus and showed post-contrast unenhanced areas suggestive of necrosis. There was evidence of blood degradation products consistent with a subacute bleed. There was stretching and compression of the optic chiasm. Features were consistent with pituitary apoplexy on an underlying macroadenoma. Humphrey’s visual field examination revealed a left visual field index (VFI) of only 1% with optic disc pallor, while the right was 64% with temporal hemianopia as shown in Fig. 3.

**Fig. 4:**
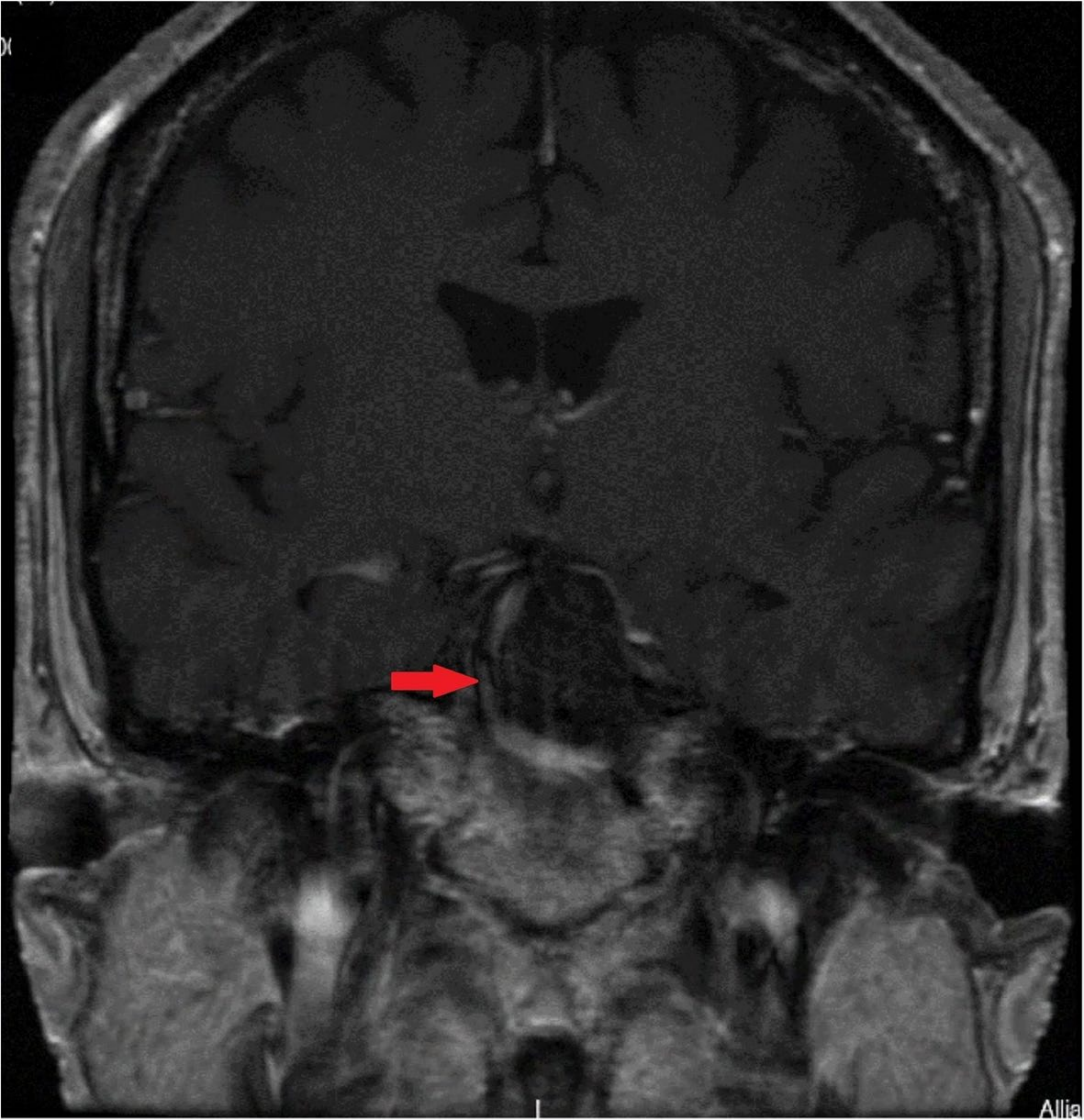
T1 coronal MRI pituitary 6 months post-hypophysectomy showing 13 mm residual tumour

Intravenous hydrocortisone was commenced and the patient urgently referred to the neurosurgical team. He underwent emergency trans-sphenoidal hypophysectomy with circumferential excision of the apoplectic macroadenoma on day seven of the presentation. Follow-up post-hypophysectomy MRI 6 months later showed optic chiasm decompression and a 13-mm residual tumour as shown in Fig. 4.

Post-trans-sphenoidal hypophysectomy, the patient recovered well and had a marked improvement in the right eye and 50% recovery of vision in the left eye. The patient was restarted on ibrutinib and venetoclax 2 weeks following surgery as it was felt that the benefit-to-risk ratio favoured continuation of the treatment and there was little pituitary tissue remaining. The patient had a pituitary MRI at three and 6 months post-operatively which showed a good surgical response.

CLL therapy was withdrawn a year later, following radiological, clinical and biochemical remission. Approximately 1 year post-CLL treatment with ibrutinib and venetoclax, the patient was in complete remission with negative bone marrow for the first time since diagnosis.

## Discussion and conclusions

Central nervous system (CNS) haemorrhage is a rare side effect of ibrutinib. A randomised phase 3 trial comparing ibrutinib and chlorambucil in the treatment of CLL assessed the risk of major haemorrhage. Of the ibrutinib arm and following a median 17.4-month exposure, 4% (6 patients) developed a major haemorrhage; only 2 (1.3%) of which were CNS haemorrhages. Those patients had other comorbidities and cardiovascular risk factors predisposing them to this [3]. A literature search revealed that this complication is a rare occurrence; there have been no reported cases of pituitary apoplexy secondary to ibrutinib therapy in the United Kingdom (UK), making this the first case of its type to be published in the UK. There was only one similar case reported in the United States of America [4]. It is not known whether the treatment was restarted for that patient. Apoplexy is a known complication of pituitary macroadenoma and a thorough assessment is required to assess the risks and benefits of using ibrutinib in patients with pituitary macroadenoma [1].

Studies have shown that ibrutinib increases the risk of bleeding [5]. Ibrutinib is a Bruton tyrosine kinase (BTK) inhibitor which causes apoptosis and reduced chemotaxis of CLL cells [5]. Ibrutinib increases the risk of bleeding through its thrombocytopenic effect as well as platelet dysfunction associated with tyrosine kinase inhibition [6, 7]. BTK is involved in platelet signalling through cell-surface receptors expressed on platelets. These mediate platelet adhesion through von Willebrand factor. By inhibiting BTK platelet aggregation is suppressed thus increasing the risk of bleeding [7]. This patient was thrombocytopenic with a nadir platelet count of 89 × 10^9^/L. A combination of thrombocytopenia along with the increased risk of bleeding associated with ibrutinib as described are likely to have caused pituitary apoplexy in this patient’s case.

Ibrutinib has Vascular Endothelial Growth Factor (VEGF) inhibition effects; a study on mice found that mice administered with OSI-930 (a tyrosine kinase receptor inhibitor including VEGF receptors 1 and 2) developed pituitary haemorrhage. All mice who received the treatment for 6 days developed pituitary haemorrhage and 66.7 of those who received it for 3 days developed this side effect. It is thought that OSI-930 disrupts the vascular supply of the pituitary gland by reducing the capillary network thus resulting in haemorrhage formation [8].

Due to the rarity of this complication, there is no clear data about the probability of developing pituitary apoplexy in CLL with or without BTK inhibitor therapy, but there have been a handful of reported cases of patients being diagnosed with CLL or chronic myeloid leukaemia after presenting with pituitary apoplexy; thought to be due to thrombocytopenia associated with the underlying malignancy [9] or presenting with a pituitary mass [10].

Research has shown that patients with trisomy 12 (as in this case) are likely to be more sensitive to ibrutinib [11]. This patient had multiple risk factors that increased his risk of bleeding, thus it is likely that the patient developed pituitary macroadenoma apoplexy as a result ibrutinib therapy.

This is a unique and rare case of pituitary macroadenoma apoplexy following the commencement of ibrutinib for the treatment of CLL. A thorough assessment is required to assess the risks and benefits of using ibrutinib in patients with pituitary macroadenoma to avoid serious complications including haemorrhage which can be fatal. Patients with suspected pituitary apoplexy should be thoroughly and rapidly investigated as time is of the essence to avoid permanent vision loss.

## Data Availability

Not applicable.

## Abbreviations

SIADH: Syndrome of Inappropriate Antidiuretic Hormone Secretion
CLL: Chronic lymphocytic leukaemia
ED: Emergency department
VFI: Visual field index
BTK: Bruton tyrosine kinase
VEGF: Vascular endothelial growth factor
